# Supplementary material for: Associations of 16-Year Population Dynamics in Range-Expanding Moths with Temperature and Years since Establishment
Source: Insects. 2023 Jan 6;14(1):55. doi: 10.3390/insects14010055 (PMC9864116; doi:10.3390/insects14010055)

insects

MDPI

Associations of 16-year population dynamics in range-expanding moths with temperature and years since establishment

Per-Eric Betzholtz[1*], Anders Forsman[1] & Markus Franzén[1]

[1] Department of Biology and Environmental Science, Linnaeus University, SE-39182 Kalmar, Sweden

[*] Correspondence: per-eric.betzholtz@lnu.se

SUPPLEMENTARY INFORMATION

Table S1. The 107 range-expanding moths arranged in taxonomic order of their family, subfamily and yearly abundance sorted taxonomically.

Family	Subfamily	Species/Year	2005	2006	2007	2008	2009	2010	2011	2012	2013	2014	2015	2016	2017	2018	2019	2020	Total
Pyralidae	Phycitinae	*Oncocera semirubella*		2	2	2	3		1	3	7	11	4	5	2	5	6	6	59
Pyralidae	Phycitinae	*Sciota fumella*							1			1		3		4		3	12
Pyralidae	Phycitinae	*Sciota adelphella*	1		2	3		12	11	2		4	1		1	4	3		44
Pyralidae	Phycitinae	*Nephopterix angustella*			3	1					3	1		2	2	50	8	1	71
Pyralidae	Pyralinae	*Pyralis regalis*													2	3	13	8	26
Crambidae	Pyraustinae	*Loxostege turbidalis*								2			1						3
Crambidae	Pyraustinae	*Loxostege sticticalis*		8	7	5	8	4	10	3	1	1		1		1	22	8	79
Crambidae	Pyraustinae	*Nascia cilialis*							3					2	2	2	1	3	13
Crambidae	Pyraustinae	*Ostrinia palustralis*	2	4	1		1								1	1	1		11
Crambidae	Pyraustinae	*Anania verbascalis*	1		8	3	4	2	15	4	15	11	4	1	2	5	11	22	108
Crambidae	Spilomelinae	*Udea ferrugalis*		1													12	5	18
Crambidae	Spilomelinae	*Udea accolalis*														19			19
Crambidae	Spilomelinae	*Diasemia reticularis*							1			1	2	1		15	5		25
Crambidae	Spilomelinae	*Nomophila noctuella*	1	38	95	1	1	105	3	12	20	87	34	5	1	50	236	8	697
Crambidae	Spilomelinae	*Antigastra catalaunalis*		1															1

Crambidae	Spilomelinae	*Palpita vitrealis*										2		2		1	2	5	12
Crambidae	Glaphyriinae	*Evergestis frumentalis*							1										1
Crambidae	Crambinae	*Euchromius ocellea*			2							1			1		3	8	15
Drepanidae	Drepaninae	*Watsonalla binaria*		1		1	5	8	45	15	49	102	45	89	46	296	51	49	802
Drepanidae	Drepaninae	*Watsonalla cultraria*												4					4
Drepanidae	Drepaninae	*Drepana curvatula*		1	2			1	1			1	1		1	3	1		12
Geometridae	Sterrhinae	*Cyclophora annularia*												1					1
Geometridae	Sterrhinae	*Cyclophora porata*				1									3	2			6
Geometridae	Sterrhinae	*Cyclophora linearia*	1	5			5	1	3		2		1	2		5	5		30
Geometridae	Sterrhinae	*Idaea ochrata*		1			2	2	1				1	1	2	6	9	4	29
Geometridae	Larentiinae	*Orthonama obstipata*	1					1							1			1	4
Geometridae	Larentiinae	*Xanthorhoe biriviata*												1	1				2
Geometridae	Larentiinae	*Catarhoe rubidata*				1						1		1		3	1		7
Geometridae	Larentiinae	*Costaconvexa polygrammata*		1						1							1		3
Geometridae	Larentiinae	*Horisme corticata*													1				1
Geometridae	Larentiinae	*Chloroclystis v-ata*	2		3	3	2	4	6		10	3	7	15	27	45	15	13	155
Geometridae	Larentiinae	*Eupithecia pulchellata*																1	1
Geometridae	Larentiinae	*Chesias legatella*	1			1							1	1	1	5	1	3	14
Geometridae	Ennominae	*Fagivorina arenaria*		1															1
Geometridae	Geometrinae	*Thetidia smaragdaria*	1							2		1	1	2	1	5		1	14
Sphingidae	Sphinginae	*Agrius convolvuli*	17	6	5	1	2	7	7	1	8		1		2	3	3		63
Sphingidae	Macroglossinae	*Hyles euphorbiae*							2										2
Sphingidae	Macroglossinae	*Hyles gallii*		11	93	16	26	70	108	30	120	56	56	34	60	22	50	32	784
Notodontidae	Thaumetopoeinae	*Thaumetopoea pinivora*			7														7
Notodontidae	Notodontinae	*Notodonta tritophus*	2	3	4		4	1	7	5	5	5	8	4	7	5	5	6	71
Notodontidae	Pygaerinae	*Clostera anachoreta*	1															1	2
Erebidae	Hypeninae	*Colobochyla salicalis*		1				1	4	4	1	1	4	6	5	1	2	2	32
Erebidae	Nygmiini	*Euproctis chrysorrhoea*		31					1	2		1		2	1			1	39
Erebidae	Lithosiini	*Pelosia muscerda*	2	5	1		6	8	8	2	4	8	32	44	22	32	18	38	230

Erebidae	Lithosiini	*Pelosia obtusa*	2	5		6		1	2	2	4	3			1	3	3	2	34
Erebidae	Lithosiini	*Lithosia quadra*		1	1	8	4	3	16	12	5	20	13	3	16	15	9	4	130
Erebidae	Lithosiini	*Eilema griseola*					1		1				4	1	1	4		3	15
Erebidae	Arctiini	*Callimorpha dominula*			1														1
Erebidae	Boletobiinae	*Eublemma purpurina*															1		1
Erebidae	Erebinae	*Minucia lunaris*		1															1
Erebidae	Erebinae	*Grammodes stolida*															1		1
Erebidae	Erebinae	*Catocala fulminea*							1	2						1			4
Erebidae	Erebinae	*Catocala fraxini*	11	35	9	3	9		30	7	8	5	8	9	8	4	11	10	167
Erebidae	Erebinae	*Catocala nupta*	3	15	18	5	5	8	47	4	13	4	9	12	15	10	12	10	190
Noctuidae	Acontiinae	*Acontia trabealis*	2	2	11	2		6	54	43	8	3	1	5		15	2	13	167
Noctuidae	Plusiinae	*Abrostola triplasia*	59	24	93	87	72	80	170	89	67	127	75	73	202	106	70	85	1479
Noctuidae	Plusiinae	*Trichoplusia ni*			1														1
Noctuidae	Plusiinae	*Macdunnoughia confusa*	4	21	35	7	12	6	15	4	13	11	6	2	10	14	17	16	193
Noctuidae	Plusiinae	*Autographa mandarina*	7	15	9		4	3	61	7		1							107
Noctuidae	Plusiinae	*Syngrapha interrogationis*	2	8		2		1	4	6	7	7	1			2		1	41
Noctuidae	Cuculliinae	*Cucullia fraudatrix*							2	1				4		1		2	10
Noctuidae	Cuculliinae	*Cucullia artemisiae*		1							1			1					3
Noctuidae	Cuculliinae	*Cucullia scrophulariae*															1	2	3
Noctuidae	Eustrotiinae	*Deltote deceptoria*		1					7	5	1								14
Noctuidae	Acronictinae	*Acronicta albovenosa*	6	12	5	23	15	9	14	7	28	48	7	8	2	98	44	41	367
Noctuidae	Condicinae	*Eucarta virgo*	4	14	26	9	9	136	138	31	25	91	24	27	18	83	17	10	662
Noctuidae	Heliothinae	*Heliothis peltigera*			1								1						2
Noctuidae	Heliothinae	*Heliothis viriplaca*		5	5	3	1	10	3			6	3			3	24	23	86
Noctuidae	Heliothinae	*Helicoverpa armigera*		3								7	7		8		4	1	30
Noctuidae	Bryophilinae	*Cryphia algae*							1			1	2	1	3	4	64	114	190
Noctuidae	Pseudeustrotiini	*Pseudeustrotia candidula*	2	7	16	7	5	11	138	14	44	87	9	11	35	35	337	92	850
Noctuidae	Prodeniini	*Spodoptera exigua*											6			4	1	1	12
Noctuidae	Caradrinini	*Hoplodrina ambigua*	2	1	3	19	94	10	16	37	33	91	587	1186	632	513	3284	1575	8083
Noctuidae	Caradrinini	*Proxenus lepigone*			1									12	3	2	18	7	43

Noctuidae	Phlogophorini	*Phlogophora meticulosa*	54	61	199	115	53	3	13	43	23	73	366	155	49	81	109	243	1640
Noctuidae	Apameini	*Hydraecia ultima*		7	5		1	3	49	4	2	12	3		1	1			88
Noctuidae	Apameini	*Sedina buettneri*							2		1	2	2	2		1		2	12
Noctuidae	Apameini	*Archanara neurica*						1											1
Noctuidae	Apameini	*Coenobia rufa*												1	1	13	16	43	74
Noctuidae	Apameini	*Photedes extrema*				1		1											2
Noctuidae	Apameini	*Globia sparganii*			1	2		3	1		4	3	7	1	1	4	8	8	43
Noctuidae	Apameini	*Globia algae*			1		1	1		1	2	7	4	2	1	6	11	4	41
Noctuidae	Xylenini	*Cirrhia gilvago*	4	11	2		1	1	2	1	10	6	1	9	2	15	4		69
Noctuidae	Xylenini	*Agrochola lychnidis*					1		1	2			1	1			1		7
Noctuidae	Xylenini	*Xylena exsoleta*												1					1
Noctuidae	Xylenini	*Cosmia affinis*								1			1				1	1	4
Noctuidae	Xylenini	*Atethmia centrago*															1	2	3
Noctuidae	Xylenini	*Blepharita amica*							1						1		1		3
Noctuidae	Hadenini	*Lacanobia splendens*							1					1		1			3
Noctuidae	Hadenini	*Conisania leineri*							1										1
Noctuidae	Leucaniini	*Mythimna turca*	9	11	2	5	4	7	12	13	24	20	19	23	35	29	6	4	223
Noctuidae	Leucaniini	*Mythimna vitellina*		4											2			1	7
Noctuidae	Leucaniini	*Mythimna albipuncta*	4	9	4	15	31	15	12		1	29	102	184	88	30	137	227	888
Noctuidae	Leucaniini	*Mythimna l-album*										2	8	2		1	4	2	19
Noctuidae	Noctuini	*Peridroma saucia*	2	1															3
Noctuidae	Noctuini	*Actebia praecox*	3	4	2	2	12	13	22	24	67	26	51	42	27	28	36	30	389
Noctuidae	Noctuini	*Agrotis bigramma*										1	1						2
Noctuidae	Noctuini	*Agrotis ripae*		3	1	1	6	6	8	2	5	7	5	5	3	37	7	16	112
Noctuidae	Noctuini	*Agrotis puta*														1	2	2	5
Noctuidae	Noctuini	*Agrotis ipsilon*	3	87	79	8	5	17	25	18	10	28	26	35	14	208	11	10	584
Noctuidae	Noctuini	*Chersotis cuprea*		1	1	1	1			2									6
Noctuidae	Noctuini	*Noctua interposita*			2		2	1	2				3	3	5	17	9	10	54
Noctuidae	Noctuini	*Noctua janthina*	103	35	17	37	71	34	62	60	136	244	58	102	62	69	24	30	1144
Noctuidae	Noctuini	*Noctua interjecta*	51	2	19	54	136	127	64	35	62	273	212	713	481	543	281	215	3268
Noctuidae	Noctuini	*Xestia alpicola*							2										2
Noctuidae	Noctuini	*Xestia ditrapezium*							1				1	1	3	6	1	1	14
Nolidae	Chloephorinae	*Nycteola asiatica*		2	1	1							3	1	1				9

Figure S1. Average ambient air temperature during May - July at the WMO meteorological station Ölands södra udde during the study period 2005 - 2020.

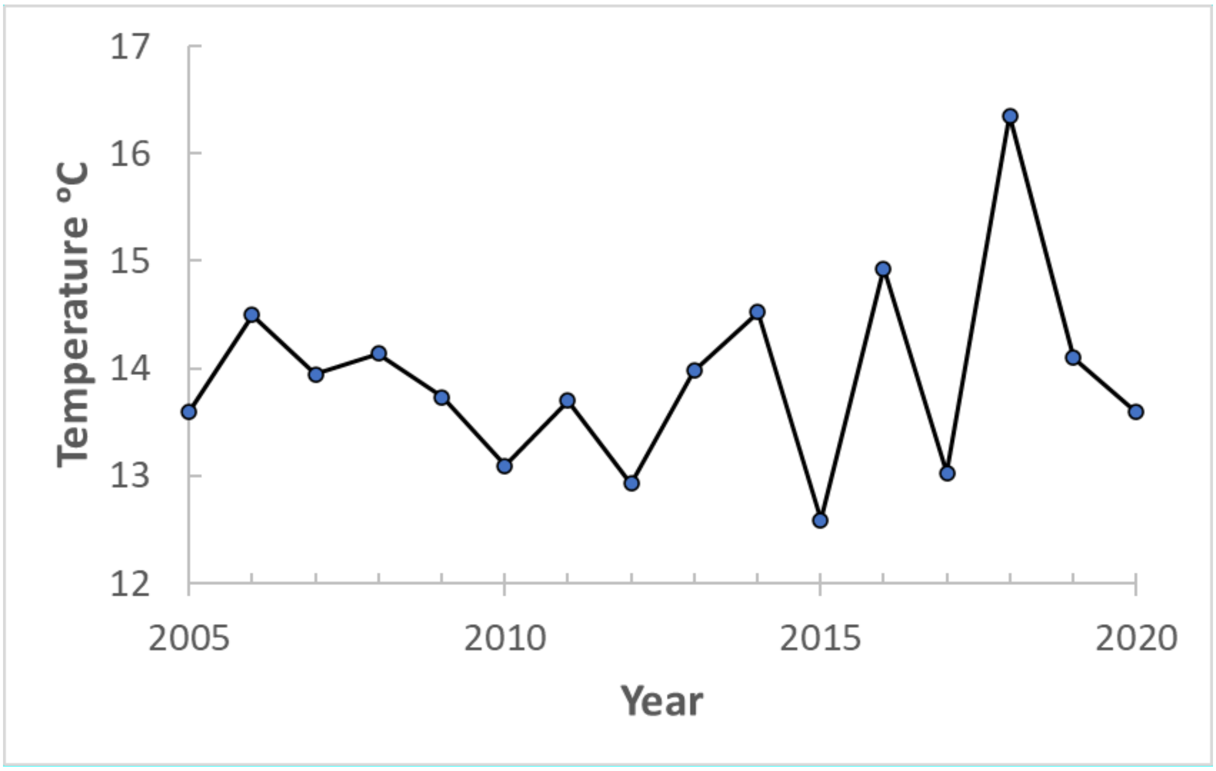

Supplement: Supplementary file 1 [file insects-14-00055-s001.zip › insects-2049597-SI.pdf]
